# Circulating plasma microRNA-126, microRNA-145, and microRNA-155 and their association with atherosclerotic plaque characteristics

**Published:** 2020-01-13

**Authors:** Evija Knoka, Karlis Trusinskis, Mairita Mazule, Ieva Briede, William Crawford, Sanda Jegere, Indulis Kumsars, Inga Narbute, Dace Sondore, Aivars Lejnieks, Andrejs Erglis

**Affiliations:** ^1^Latvian Centre of Cardiology, Pauls Stradins Clinical University Hospital, Riga, LV-1002, Latvia; ^2^Department of Internal Diseases, Riga Stradins University, Riga, LV-1007, Latvia; ^3^Faculty of Medicine, University of Latvia, Riga, LV-1050, Latvia; ^4^Department of Endocrinology and Internal Medicine, Riga East Clinical University Hospital, Riga, LV-1038, Latvia; ^5^Faculty of Medicine, University of Oxford, Oxford OX1 2HB, United Kingdom

**Keywords:** atherosclerosis, coronary artery disease, intravascular ultrasound, microRNA

## Abstract

**Aims:**

Circulating microRNAs (miRNAs) have been identified as biomarkers for several diseases. Dysregulation of miRNA-126, microRNA-145, and microRNA-155 has been shown to be associated with atherosclerotic lesion formation. The aim of this study was to evaluate the association between atherosclerosis-related miRNAs and unfavorable atherosclerotic plaque characteristics.

**Methods and Results:**

Forty patients with stable coronary artery disease admitted for elective percutaneous coronary intervention (PCI) were enrolled in a prospective study. After PCI, intravascular ultrasound (IVUS), and iMAP-IVUS analysis were performed to assess the proportion of fibrotic, necrotic, lipidic, and calcific tissue within atherosclerotic plaques. Total RNA was isolated from plasma to evaluate the expression of circulating miRNA-126, miRNA-145, and miRNA-155. Plasma lipid and glucose metabolism-related variables were measured to determine any association with plaque characteristics or miRNA expression. Expression of miRNA-126 was negatively correlated with plaque fibrotic tissue (*r*=−0.28; *P*=0.044), while positively correlated with plaque necrotic tissue (*r*=0.31; P=0.029) and necrolipidic tissue (*r*=0.31; *P*=0.031). MiRNA-145 was positively correlated with plaque lipidic (*r*=0.32; *P*=0.023) and necrolipidic tissue (*r*=0.31; *P*=0.029). Patient age was associated with plaque fibrotic tissue (*r*=-0.41; *P*=0.005), necrotic tissue (*r*=0.33; *P*=0.022), and lipid content (*r*=0.33; *P*=0.022). High-density lipoprotein cholesterol was positively correlated with plaque necrotic (*r*=0.28; *P*=0.042) and calcific (*r*=0.28; *P*=0.044) tissue volume. Calcific tissue volume was positively correlated with C-peptide (*r*=0.34; *P*=0.033). After multivariate logistic regression analysis, both miRNA-126 and miRNA-145 expressions were associated with increased necrolipidic tissue content (β=0.34; *P*=0.050; and *β*=0.35; *P*=0.037, respectively).

**Conclusions:**

Expressions of miRNA-126 and miRNA-145 were associated with increased plaque necrolipidic tissue content.

**Relevance for Patients:**

Although further research is needed to support the study data, miRNA-126 and miRNA-145 may serve as potential plaque vulnerability biomarkers in the future.

## 1. Introduction

MicroRNAs (miRNAs) are small non-coding single-stranded RNA molecules involved in post-transcriptional regulation of gene expression. MiRNAs play an important role in the cell cycle, immune modulation, metabolic processes, and stem cell differentiation [1]. Variable miRNA expression has been observed in several diseases, and they have therefore been extensively studied as biomarkers and therapeutic targets. Since miRNAs contain small fixed sequences, they represent potential targets for potent oligonucleotide-targeted anti-miRNA drugs [1]. Several circulating miRNAs associated with atherosclerosis development have been studied as potential cardiovascular disease biomarkers. In this study, we investigated the clinical relevance of three vascular miRNAs (miRNA-126, miRNA-145, and miRNA-155) which have previously shown a strong association with atherosclerosis development.

MiRNA-126 is considered to be atheroprotective, located in an intron of the epidermal growth factor-like-domain-7 gene (*EGFL7*) [2-4]. MiRNA-126 is specifically expressed in endothelial cells, modulating their phenotype and their response to migration induced by vascular endothelial growth factor and fibroblast growth factor-2. MiRNA-126 also plays an important role in regulating the expression of vascular cell adhesion molecule-1, which mediates leukocyte-endothelial cell adhesion in the arterial endothelium. Apoptotic endothelial cells in atherosclerotic lesions release miRNA-126-containing apoptotic bodies, which attracts endothelial progenitor cells to the site to prevent lesion development [3]. Atheroprotective miRNA-145 is expressed in vascular smooth muscle cells (VSMC) and has a crucial role in regulating cell differentiation. Overexpression of miRNA-145 upregulates the expression of genes involved in VSMC differentiation, promoting differentiation of VSMCs into their contractile phenotype [5]. Deficiency of miRNA-145 results in inappropriate VSMC plasticity and promotes the phenotypic switch from a contractile to a proliferative and migratory state [4], therefore enhancing atherosclerotic lesion formation. MiRNA-155 has numerous functions, including roles in hematopoietic cell differentiation, immunity, vascular remodeling [6], and inflammatory signaling pathways. Results regarding the role of miRNA-155 on macrophages and foam cells in atherosclerotic plaques are conflicting, as both pro- and anti-inflammatory effects have been reported [4]. MiRNA-155 may enhance inflammation by promoting monocyte recruitment to atherosclerotic plaques [7] and impairing cholesterol efflux [7,8], thereby stimulating atherosclerosis progression. On the contrary, other studies have shown that miRNA-155 may be a part of a negative feedback loop in the atherosclerotic pathophysiological processes. Increased miRNA-155 expression in atherosclerotic lesions may reduce inflammation [9], while reduced expression causes atherosclerotic plaque instability and promotes atherogenesis [10].

This study evaluated the association between atherosclerosis-related circulating miRNA-126, miRNA-145, miRNA-155 expression, and coronary plaque characteristics and vulnerability, assessed by iMAP intravascular ultrasound (IVUS). We hypothesized that miRNA expression will be associated with plaque tissue types in iMAp-IVUS imaging. iMap™ IVUS (iMap; Boston Scientific Corp, Fremont, CA, USA) is a relatively new software package for assessing plaque composition (fibrotic, lipidic, necrotic, and calcific) according to backscattered ultrasound frequency spectrum [11,12]. IVUS has the ability to reliably characterize plaque tissue components with a high level of confidence. An *ex vivo* study in which a database of approximately 12,000 image regions of interest was analyzed using IVUS imaging demonstrated accuracies at the highest level of confidence as: 97%, 98%, 95%, and 98% for necrotic, lipidic, fibrotic, and calcified regions, respectively, compared to histological findings [11]. In addition, good agreement with histology was shown in an *in vivo* swine animal model [11]. In parallel, it is known that impaired lipid and glucose regulation may also negatively influence the course of atherosclerosis. We therefore sought to evaluate the association of metabolic markers with miRNA expression and plaque vulnerability, to determine whether any of these parameters differ according to the severity of coronary artery disease (CAD).

## 2. Patients and Methods

### 2.1. Patients

In a prospective single-center study, consecutive patients with stable CAD undergoing elective percutaneous coronary intervention (PCI) from December 2016 to December 2017 were screened for the study. Inclusion criteria were met and informed consent was signed in 40 patients who were enrolled in the study. The study protocol was approved by an institutional review board. The study protocol is registered in ClinicalTrials.gov database (NCT02744976). This study was performed in compliance with the Declaration of Helsinki and patients were included after a signed informed consent was obtained. The inclusion criteria were age ≥18 and <75 years in patients referred to PCI due to stable CAD in an artery suitable for IVUS pullback, and impaired glucose regulation defined as glycated hemoglobin (HbA1c) 5.7-6.4% [13]. Patients were excluded from the study in the presence of cardiac or non-cardiac illness with life expectancy of <2 years; failure to advance the IVUS catheter through the culprit lesion; acute coronary syndrome; congestive heart failure NYHA III-IV; diabetes mellitus; glucose lowering therapy; significant renal disease defined as glomerular filtration rate <30 ml/min/m^2^; previous PCI in the target vessel; and heavily calcified vessels. A fasting blood sample was obtained from all participants to determine plasma glucose, HbA1c, C-peptide, serum total cholesterol, triglyceride, high-density lipoprotein cholesterol, and low-density lipoprotein cholesterol (LDL-C) levels. The patient’s baseline demographic, clinical characteristics and procedural data were collected. The severe CAD was defined as luminal stenosis >50% in three coronary arteries with or without left main involvement, determined by quantitative coronary angiography.

### 2.2. Evaluation of miRNA expression

To isolate plasma miRNA, fasting venous blood samples were taken in ethylenediaminetetraacetic acid -containing tubes from patients before heparin administration and cardiac catheterization. Plasma was isolated with rapid centrifugation for 20 min at 4°C, following which the supernatant was stored at –80°C in RNAse/DNAse-free tubes. Before nucleic acid purification, samples were thawed at room temperature (15-25°C). Total RNA (containing miRNA) was purified according to the miRNeasy Serum/Plasma kit protocol (Qiagen, Valencia, CA). Lyophilized *Caenorhabditis elegans* miRNA-39 (cel-miRNA-39) was used as a spike-in control during the RNA purification process. After purification, 10 ng of total RNA was used to perform miRNA-126, miRNA-145, miRNA-155, and cel-miRNA-39 reverse transcription using TaqMan MicroRNA (miRNA) Reverse Transcription Kits (Applied Biosystems, CA, USA). Real-time polymerase chain reaction (RT-PCR) was used to detect miRNA-126, miRNA-145, and miRNA-155 expression. MiRNA amplification was performed using TaqMan miRNA assay kits (Applied Biosystems). For further data analysis, the relative expression levels of miRNAs were calculated using the comparative delta *Ct* (threshold cycle number) method (2-ΔΔCT) implemented in the RT-PCR System software. The relative *Ct* for cel-miRNA-39 was used as a control in the normalization of miRNA-126, miRNA-145, and miRNA-155 *Ct*.

### 2.3. Plaque tissue characterization with iMAP-IVUS.

**Figure 1:**
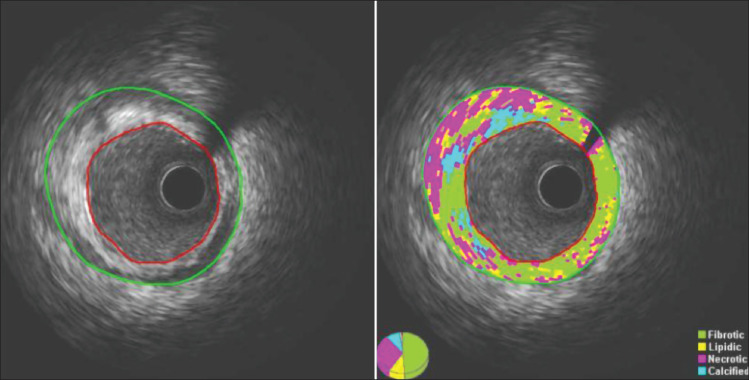
Plaque tissue characterization with iMap software (QIvus 2.0; Medis medical imaging systems, Leiden, The Netherlands).

All PCI procedures were performed according to procedure guidelines. After successful treatment of the culprit lesion, an ultrasound imaging catheter (AtlantisTM SR Pro; 40 MHz, mechanical-type transducer, 3.2 F, Boston Scientific Corporation, Natick, MA, USA) was advanced >15 mm beyond the culprit lesion, followed by motorized transducer pullback at 0.5 mm/s. The IVUS data were stored on a hard disk for off-line analysis. Quantitative analysis of gray-scale IVUS images was performed according to the criteria of the American College of Cardiology Clinical Expert Consensus Document on IVUS [14]. The segment of interest during IVUS analysis was defined as the 10 mm long segment at least 5 mm proximal to the stent with the greatest plaque burden. In exceptional cases, when the proximal segment could not be analyzed due to a proximal culprit lesion, a segment distal to the stent was analyzed. Plaque tissue analysis of a non-culprit segment was performed as patients will be followed up with further plaque tissue characterization at 2 years, to monitor for tissue changes. Virtual plaque assessment was performed with iMap software (QIvus 2.0; Medis medical imaging systems, Leiden, The Netherlands) to determine the percentage of fibrotic, lipidic, necrotic, and calcific tissue present (Figure 1). Criteria for plaque vulnerability included high lipidic, necrotic and calcific tissue content, and low fibrotic tissue content. Increased size of lipid-rich necrotic plaque tissue is believed to be a risk factor for atherosclerotic plaque rupture; therefore, a combination of lipidic and necrotic tissue was determined and defined as necrolipidic tissue and used in further analysis.

### 2.4. Statistical analysis

Data were expressed as mean±standard deviation (SD) for continuous variables or as percentages and frequencies for categorical variables. Comparisons with categorical variables were analyzed using Fisher’s exact test. Univariate logistic regression analysis was performed to examine the correlation between iMAP-IVUS tissue percentages, miRNA expression, and laboratory data parameters. Results with *P*<0.10 were included in the multivariate logistic regression analysis to determine independent factors that correlate with plaque tissue characteristics. Results were considered statistically significant if *P*<0.05. All statistical analysis was performed using PSPP 0.8.2 software.

## 3. Results

Patient baseline characteristics are shown in Table 1. The mean patient age was 61.8 years. Almost half of the patients (47.5%) had early atherosclerosis. Despite 97.5% of patients reporting statin use and 90.0% of them being on high-intensity statin treatment, most of the patients (85.0%) had unfavorable blood lipid levels. Only 47.5% of patients had reached the target LDL-C level of <1.8 mmol/L. Grayscale IVUS and iMAP-IVUS characteristics of the segment of interest are shown in Table 2.

**Table 1:** Baseline characteristics.

Characteristic	Total (*n*=40)
Age (years), mean±SD	61.8±7.89

Male sex, %	67.5

Weight (kg), mean±SD	91.50±21.16

Risk factors	

Hypertension, %	97.5

Current smoking, %	40.0

Dyslipidemia, %	85.0

Early atherosclerosis, %	47.5

Laboratory values	

LDL-C (mmol/L), mean±SD	2.15±0.94

LDL-C <1.8 mmol/L, %	47.5%

HDL-C (mmol/L), mean±SD	1.25±0.37

Triglycerides (mmol/L), mean±SD	1.58±0.79

Total cholesterol (mmol/L), mean±SD	4.07±1.05

Creatinine (mkmol/L), mean±SD	74.93±12.60

Fasting blood glucose (mmol/L), mean±SD	5.20±0.54

HbA1c (%), mean±SD	5.93±0.29

C-peptide (ng/mL), mean±SD	2.42±1.06

Statin use, %	97.5

High-intensity statin use, %	90.0

Previous myocardial infarction, %	45.0

Previous stroke, %	4.7

Previous PCI, %	57.5

Previous CABG, %	0.0

Peripheral artery disease, %	10.0

Congestive heart failure, %	66.7

Structural valve disease, %	2.5

Atrial fibrillation, %	20.0

Severe CAD, %	65.0

Total miRNA expression	

cel-miRNA-39	28.01±1.86

miRNA-126	31.30±1.47

miRNA-145	34.11±1.97

miRNA-155	34.57±2.52

Relative miRNA expression^	

miRNA-126	3.30±1.67

miRNA-145	6.11±1.42

miRNA-155	6.50±3.01

CABG: Coronary artery bypass grafting, CAD: Coronary artery disease, HDL-C: High-density lipoprotein cholesterol, IVUS: Intravascular ultrasound, LDL-C: Low-density lipoprotein cholesterol, PCI: Percutaneous coronary intervention, SD: Standard deviation. ^Relative miRNA expression is defined as an expression after normalization with cel-miRNA-39 expression

**Table 2:** Characteristics of the segment of interest in IVUS imaging.

Characteristic	Total (*n*=40)	
LM/LAD/LCX/RCA (%)	22.58/70.27/13.51/16.22	

Length (mm), mean±SD	10.32±2.93	

Lumen area (mm^2^), mean±SD	8.14±2.85	

Vessel area (mm^2^), mean±SD	15.52±5.02	

Vessel volume (mm^3^), mean±SD	155.53±59.24	

Lumen volume (mm^3^), mean±SD	82.92±37.82	

Plaque volume (mm^3^), mean±SD	72.68±32.81	

	**Mm^3^, mean±SD**	**%, mean±SD**

Fibrotic tissue (mm^3^), mean±SD	35.78±15.44	70.33±14.55

Lipid tissue (mm^3^), mean±SD	6.11±5.75	9.39±5.38

Necrotic tissue (mm^3^), mean±SD	10.90±9.25	17.98±9.68

Calcific tissue (mm^3^), mean±SD	1.31±1.17	2.50±2.10

Necrolipidic tissue (%), mean±SD	17.01±9.25	27.27±13.90

LAD: Left anterior descending coronary artery, LCX: Left circumflex coronary artery, LM: Left main coronary artery, RCA: Right coronary artery. SD: Standard deviation, IVUS: Intravascular ultrasound

Association of laboratory and patient characteristics with plaque tissue content is shown in Table 3. Increased age was associated with unfavorable plaque tissue characteristics. Higher lipidic (*r*=0.33; *P*=0.022), necrotic (*r*=0.33; *P*=0.022), and lower fibrotic (*r*=−0.41; *P*=0.005) tissue content were observed in older patients. The presence of severe CAD was not associated with any laboratory parameter or plaque tissue characteristic.

**Table 3:** Association between plaque tissue characteristics and laboratory values.

Characteristic	Fibrotic		Lipidic		Necrotic		Necrolipidic		Calcific	
				
	*r*	*P*	*r*	*P*	*r*	*P*	*r*	*P*	*r*	*P*
Age	−0.41	**0.005**	0.33	**0.022**	0.33	**0.022**	0.38	0.10	0.22	0.93

Weight	0.16	0.174	−0.14	0.197	−0.16	0.17	−0.19	0.132	0.04	0.396

Total cholesterol	−0.06	0.364	−0.09	0.29	0.02	0.461	0.0	0.493	0.13	0.226

LDL-C	−0.09	0.288	0.03	0.431	−0.01	0.485	0.02	0.46	0.01	0.478

HDL-C	−0.21	0.098	−0.01	0.47	0.28	**0.042**	0.2	0.115	0.28	**0.044**

Triglycerides	0.24	0.07	−0.19	0.129	−0.25	0.062	−0.23	0.08	−0.06	0.35

Glucose	0.02	0.441	−0.23	0.083	0.05	0.391	−0.12	0.244	−0.02	0.46

HbA1c	0.18	0.142	−0.29	**0.043**	−0.17	0.158	−0.25	0.068	0.07	0.331

C-peptide	−0.02	0.46	−0.29	0.059	0.10	0.308	−0.1	0.299	0.34	**0.033**

HDL-C: High-density lipoprotein cholesterol, LDL-C: Low-density lipoprotein cholesterol, *r*: Pearson correlation coefficient, *P*:** Value of significance, ^Significant values are marked in bold

**Figure 2:**
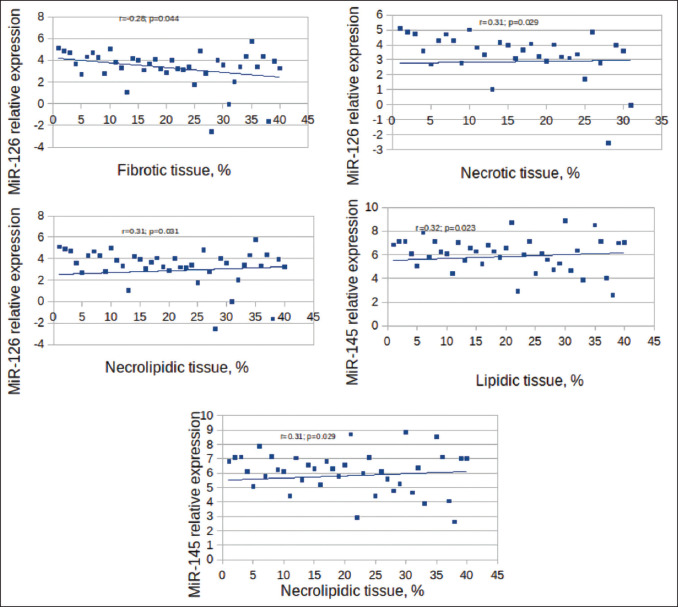
Significant associations between miRNA relative expression and plaque tissue types. Univariate logistic analysis in 40 patients shows significant correlations between miRNA-126 and fibrotic, necrotic, necrolipid tissue, and miRNA-145 expression and plaque lipidic and necrolipidic tissue.

Correlation of miRNA expression with a plaque and patient parameters is depicted in Table 4. MiRNA-126 and miRNA-145 expression had a moderate correlation with plaque tissue characteristics. As shown in Figure 2, miRNA-126 expression negatively correlated with plaque fibrotic tissue content (*r*=−0.28; *P*=0.044), positively with necrotic (*r*=0.31; *P*=0.029), and necrolipidic (*r*=0.31; *P*=0.031) tissue content. Higher miRNA-145 expression was associated with increased plaque lipidic (*r*=0.32; *P*=0.023) and necrolipidic (*r*=0.31; *P*=0.029) tissue content. Although miRNA-155 expression was not significantly associated with plaque tissue characteristics, its expression correlated with several metabolic parameters (Table 4). We did not find any association between miRNA expression and the severity of CAD.

**Table 4:** Association between miRNA expression, plaque, and patient characteristics.

Characteristic	MiRNA-126		MiRNA-145		MiRNA-155	
		
	*r*	*P*	*r*	*P*	*r*	*P*
Age	0.25	0.061	0.1	0.269	−0.35	**0.014**

Weight	0.02	0.44	0.16	0.165	0.4	**0.006**

Total cholesterol	0.21	0.098	−0.18	0.139	0.01	0.479

LDL-C	0.1	0.264	−0.2	0.107	−0.17	0.152

HDL-C	0.18	0.127	−0.21	0.101	0.11	0.247

Triglycerides	0.21	0.102	0.25	0.059	0.32	**0.025**

Glucose	0.02	0.463	−0.21	0.094	0.28	**0.04**

HbA1c	−0.08	0.307	−0.2	0.115	0.1	0.27

C-peptide	−0.01	0.488	−0.22	0.116	0.45	**0.005**

Fibrotic tissue	−0.28	**0.044**	−0.26	0.06	−0.04	0.412

Lipidic tissue	0.21	0.102	0.32	**0.023**	−0.16	0.174

Necrotic tissue	0.31	**0.029**	0.22	0.092	0.1	0.282

Calcific tissue	−0.02	0.452	0.03	0.436	0.19	0.127

Necrolipidic tissue	0.31	**0.031**	0.31	**0.029**	0.01	0.47

Plaque volume	0.25	0.068	−0.06	0.363	0.12	0.239

HDL-C: High-density lipoprotein cholesterol, LDL-C: Low-density lipoprotein cholesterol,* r* Pearson correlation coefficient, *P*: value of significance, ^Significant values are marked in bold

After multivariate logistic regression analysis (Table 5), a significant correlation remained between necrolipidic tissue and both miRNA-126 (*β*=0.34; *P*=0.050) and miRNA-145 (*β*=0.37; *P*=0.037).

**Table 5:** Association between miRNA expression, patient, and laboratory characteristics in multivariate regression analysis.

Characteristic	Standard regression coefficient	T statistic	*P* value
Fibrotic tissue			

miRNA-126	−0.19	−1.17	0.249

miRNA-145	−0.23	−1.46	0.154

HDL-C	−0.06	−0.35	0.730

Age	−0.32	−1.92	0.064

Lipidic tissue			

miRNA-145	0.34	1.38	0.201

Fasting blood glucose	0.52	1.58	0.149

HbA1c	−0.41	−1.65	0.132

C-peptide	−0.40	−1.24	0.246

Age	0.39	1.38	0.201

Necrotic tissue			

miRNA-126	0.31	1.71	0.079

Triglycerides	−0.24	−1.30	0.202

HDL-C	0.10	0.56	0.579

Age	0.13	0.75	0.461

Necrolipidic tissue			

miRNA-126	0.34	2.03	0.050

miRNA-145	0.35	2.17	0.037

Triglycerides	−0.27	−1.66	0.107

HbA1c	−0.13	−0.78	0.443

HDL-C: High-density lipoprotein cholesterol, *P*: Value of significance

## 4. Discussion

In this study, we demonstrated an association of miRNA-126 and miRNA-145 expression with atherosclerotic plaque tissue characterized by iMAP-IVUS. Increased miRNA-126 expression was associated with higher necrotic and necrolipidic plaque tissue content, and lower fibrotic tissue percentage in the segment of interest. In addition, increased miRNA-145 was associated with higher plaque lipidic and necrolipidic tissue percentage and had a tendency toward correlation with lower plaque fibrotic tissue content. The association between these miRNAs and characteristics of increased plaque vulnerability was confirmed with multivariate regression analysis, with both miRNA-126 and miRNA-145 significantly associated with increased plaque necrolipidic tissue.

Our results are consistent with a previous study where miRNA-126 expression was found to be upregulated in plasma microparticles from patients with unstable angina, compared to the control group and patients with stable angina [15]. Moreover, the unstable plaque phenotype (determined by the presence of thin cap fibroatheromas in optical coherence tomography) was associated with significantly higher transcoronary gradients for miRNA-126-3p, miR126-5p, and miRNA-145-5p [16]. However, some previous studies have shown that the expression of miRNA-126 [17,18], miRNA-145 [17,19,20], and miRNA-155 [17,19] is downregulated in patients with CAD, compared to control group of non-CAD patients. MiRNA-126 [3,4], miRNA-145 [4], and miRNA-155 [4] have previously demonstrated atheroprotective properties; therefore, the connection between atherosclerosis development and downregulation of these miRNAs is plausible. Our findings however contradict this hypothesis, as our results demonstrated that increased miRNA-126 and miRNA-145 expression levels were associated with a more vulnerable plaque phenotype. Two explanations for this finding are possible. First, it could suggest that the studied miRNAs have a variable role in coronary atherogenesis depending on the stage of the disease. While the upregulation of these miRNAs may be atheroprotective in the early phases of plaque development, higher miRNA expression could serve as a promoter of plaque destabilization later on. Second, miRNA-126 and miRNA-145 upregulation could be a part of a negative feedback mechanism. MiRNA-126 and miRNA-145 expression would therefore be increased in the setting of vulnerable atheromatous plaque, to prevent the plaque from further destabilization. Consequently, inconsistency in our and previous study results indicate that further studies are needed to provide more evidence for these miRNAs and their role in plaque pathogenesis and their potential use as plaque vulnerability biomarkers.

The expression of miRNA-155 did not show any significant correlation with plaque tissue characteristics in our study. MiRNA-155 was however the only miRNA that was associated with several metabolic parameters of the study group. MiRNA-155 was positively correlated with fasting blood glucose, triglycerides, C-peptide, and patient weight; and negatively correlated with patient age. On multivariate analysis, a significant correlation remained only between miRNA-155 and C-peptide (*β*=0.49; *P*=0.027) (data not are shown). These results suggest that miRNA-155 may play a role in the development of insulin resistance. This finding is consistent with a study evaluating the role of miRNA-155 in mice, in which the global transgenic overexpression of miRNA-155 led to improved glucose tolerance, glucose uptake in cells and insulin sensitivity [21]. Another study showed that glucose metabolism and the adaptation of pancreatic β-cells to obesity-induced insulin resistance were improved after miRNA-155-5p upregulation as a response to hyperlipidemia-associated endotoxemia [22]. Consequently, our data suggest that in the presence of elevated fasting blood glucose, C-peptide, and triglycerides; miRNA-155 may be upregulated to prevent further progression of insulin resistance.

Our study revealed that impaired glucose regulation has a somewhat minimal impact on plaque tissue characteristics. C-peptide had a positive correlation with plaque calcific tissue content, while HbA1c had a negative correlation with plaque lipidic tissue content. However, these associations were not present after the multivariate analysis. These findings are inconsistent with the previous studies, where it has been shown that high HbA1c was associated with a lipid-rich necrotic core determined by magnetic resonance imaging [23]. Moreover, IVUS imaging studies have shown that impaired glucose regulation is associated with coronary artery remodeling [24] and coronary plaque vulnerability [25,26] with more lipid-rich plaque content and decreased plaque fibrous volume [26,27]. It is possible that our study failed to corroborate this association between impaired glucose regulation and unfavorable plaque characteristics due to the small study sample and lack of control group.

### 4.1. Limitations

The sample size was relatively small; therefore, the results of this study should be interpreted with caution. We did not measure transcoronary miRNA gradients; therefore, miRNA expression presented in this study could be altered by other vascular beds. This study lacked a control group of normal glucose regulation patients. Pre-PCI culprit lesion was not analyzed. It may be speculated that stronger associations of miRNA expression could be observed for the culprit lesion. However, non-culprit segment for plaque tissue analysis was chosen due to the fact that patients will be followed up and plaque tissue characterization will be done at 2 years follow-up to determine tissue changes in the target, non-stented segment. In some cases, the segment of interest proximal to the stent could not be analyzed. miRNA expression correlates with qualitative but not quantitative plaque characteristics in patients with CAD.

## 5. Conclusion

Increased expression of miRNA-126 and miRNA-145 was associated with increased plaque necrolipidic tissue content, and therefore vulnerable atherosclerotic plaque tissue characteristics by iMAP-IVUS. miRNAs and plaque tissue characteristics did not differ in the groups of patients according to CAD severity. Further studies are needed to provide more evidence for these miRNAs as potential plaque vulnerability biomarkers.

## References

[1] Christopher AF, Kaur RP, Kaur G, Kaur A, Gupta V, Bansal P (2016). MicroRNA Therapeutics:Discovering Novel Targets and Developing Specific Therapy. Perspect Clin Res.

[2] Qu QQ, MJ MM, Pan JJ, Shi XJ, Zhang ZJ, Tang YH, Yang GY MicraRNA-126 is a Prospective Target for Vascular Disease. Neuroimmunol Neuroinflammation.

[3] Raitoharju E, Oksala N, Lehtimaki T (2013). MicroRNAs in the Atherosclerotic Plaque. Clin Chem.

[4] Andreou I, Sun X, Stone PH, Edelman ER, Feinberg MW (2015). miRNAs in Atherosclerotic Plaque Initiation, Progression, and Rupture. Trends Mol Med.

[5] Zhao W, Zhao SP, Zhao YH (2015). MicroRNA-143/-145 in Cardiovascular Diseases. Biomed Res Int.

[6] Cao RY, Li Q, Miao Y, Zhang Y, Yuan W, Fan L (2016). The Emerging Role of MicroRNA-155 in Cardiovascular Diseases. Biomed Res Int.

[7] Nazari-Jahantigh M, Wei Y, Noels H, Akhtar S, Zhou Z, Koenen RR (2012). MicroRNA-155 Promotes Atherosclerosis by Repressing Bcl6 in Macrophages. J Clin Invest.

[8] Du F, Yu F, Wang Y, Hui Y, Carnevale K, Fu M (2014). MicroRNA-155 Deficiency Results in Decreased Macrophage Inflammation and Attenuated Atherogenesis in Apolipoprotein E-Deficient Mice. Arterioscler Thromb Vasc Biol.

[9] Zhu J, Chen T, Yang L, Li Z, Wong MM, Zheng X (2012). Regulation of microRNA-155 in Atherosclerotic Inflammatory Responses by Targeting MAP3K10. PLoS One.

[10] miR155 Deficiency Enhances Atherosclerosis and Decreases Plaque Stability in Hyperlipidemic Mice (2012). PLoS One.

[11] Sathyanarayana S, Carlier S, Li W, Thomas L (2009). Characterisation of Atherosclerotic Plaque by Spectral Similarity of Radiofrequency Intravascular Ultrasound Signals. EuroIntervention.

[12] Shin ES, Garcia-Garcia HM, Ligthart JM, Witberg K, Schultz C, van der Steen AF (2011). *In vivo* Findings of Tissue Characteristics Using iMap™IVUS and Virtual Histology™IVUS. EuroIntervention.

[13] American Diabetes Association. Diagnosis and Classification of Diabetes Mellitus (2010). Diabetes Care.

[14] Mintz GS, Nissen SE, Anderson WD, Bailey SR, Erbel R, Fitzgerald PJ (2001). American College of Cardiology Clinical Expert Consensus Document on Standards for Acquisition, Measurement and Reporting of Intravascular Ultrasound Studies (IVUS). A Report of the American College of Cardiology Task Force on Clinical Expert Consensus Documents. J Am Coll Cardiol.

[15] Ren J, Zhang J, Xu N, Han G, Geng Q, Song J (2013). Signature of Circulating microRNAs as Potential Biomarkers in Vulnerable Coronary Artery Disease. PLoS One.

[16] Leistner DM, Boeckel JN, Reis SM, Thome CE, De Rosa R, Keller T (2016). Transcoronary Gradients of Vascular miRNAs and Coronary Atherosclerotic Plaque Characteristics. Eur Heart J.

[17] Fichtlscherer S, De Rosa S, Fox H, Schwietz T, Fischer A, Liebetrau C (2010). Circulating microRNAs in Patients with Coronary Artery Disease. Circ Res.

[18] Li HY, Zhao X, Liu YZ, Meng Z, Wang D, Yang F (2016). Plasma microRNA-126-5p is Associated with the Complexity and Severity of Coronary Artery Disease in Patients with Stable Angina Pectoris. Cell Physiol Biochem.

[19] Faccini J, Ruidavets JB, Cordelier P, Martins F, Maoret JJ, Bongard V (2017). Circulating miR-155, miR-145 and let-7c as Diagnostic Biomarkers of the Coronary Artery Disease. Sci Rep.

[20] Gao H, Guddeti RR, Matsuzawa Y, Liu LP, Su LX, Guo D (2015). Plasma Levels of microRNA-145 are Associated with Severity of Coronary Artery Disease. PLoS One.

[21] Lin X, Qin Y, Jia J, Lin T, Lin X, Chen L (2016). MiR-155 Enhances Insulin Sensitivity by Coordinated Regulation of Multiple Genes in Mice. PLoS Genet.

[22] Zhu M, Wei Y, Geißler C, Abschlag K, Corbalán Campos J, Hristov M (2017). Hyperlipidemia-induced microRNA-155-5p Improves β-cell Function by Targeting Mafb. Diabetes.

[23] Sun B, Zhao H, Liu X, Lu Q, Zhao X, Pu J (2016). Elevated Hemoglobin A1c is Associated with Carotid Plaque Vulnerability:Novel Findings from Magnetic Resonance Imaging Study in Hypertensive Stroke Patients. Sci Rep.

[24] Kim SH, Moon JY, Lim YM, Kim KH, Yang WI, Sung JH (2015). Association of Insulin Resistance and Coronary Artery Remodeling:An Intravascular Ultrasound Study. Cardiovasc Diabetol.

[25] Iguchi T, Hasegawa T, Otsuka K, Matsumoto K, Yamazaki T, Nishimura S (2014). Insulin Resistance is Associated with Coronary Plaque Vulnerability:Insight from Optical Coherence Tomography Analysis. Eur Heart J Cardiovasc Imaging.

[26] Mitsuhashi T, Hibi K, Kosuge M, Morita S, Komura N, Kusama I (2011). Relation between Hyperinsulinemia and Nonculprit Plaque Characteristics in Nondiabetic Patients with Acute Coronary Syndromes. JACC Cardiovasc Imaging.

[27] Amano T, Matsubara T, Uetani T, Nanki M, Marui N, Kato M (2008). Abnormal Glucose Regulation is Associated with Lipid-rich Coronary Plaque:Relationship to Insulin Resistance. JACC Cardiovasc Imaging.

